# Multifractal Nonlinearity in Behavior During a Computer Task with Increasing Difficulty: What Does It Teach Us?

**DOI:** 10.3390/e27080843

**Published:** 2025-08-08

**Authors:** Alix Bouni, Laurent M. Arsac, Olivier Chevalerias, Veronique Deschodt-Arsac

**Affiliations:** 1 University of Bordeaux, CNRS, Laboratoire IMS, (Intégration du Matériau au Système), UMR 5218, F-33400 Talence, France; laurent.arsac@u-bordeaux.fr (L.M.A.); veronique.arsac@u-bordeaux.fr (V.D.-A.); 2 Centre Aquitain des Technologies de l’Information et Electroniques, F-33400 Talence, France; o.chevalerias@catie.fr

**Keywords:** multifractal nonlinearity, cognition, dynamical systems, multiplicative cascades, motor behavior

## Abstract

The complex systems approach to cognitive–motor processing values multifractal nonlinearity as a key formalism in understanding internal interactions across multiple scales that preserve adequate task-directed behaviors. By using a computer task with increasing difficulty, we focused on the potential link between the difficulty threshold during a task, assessed by the individual’s score ceiling, and the corresponding level of multifractal nonlinearity in movement behavior, assessed based on a time series of cursor displacements. Entropy-based multifractality (MF) and multifractal nonlinearity obtained using a *t*-test comparison between the original and linearized surrogate series (t**_MF_**) of the time series characterized individual adaptive capacity. A time-varying increase in the score helped in assessing performance when facing increasing difficulty. Twenty-one participants performed a herding task (7 min), which involves keeping three moving sheep near the center of a screen by controlling the mouse pointer as a repelling shepherd dog. The more the score increased, the more the increased herd movement amplitude amplified task difficulty. The time course of the score, score dynamics (score-dyn), markedly diverged across participants, exhibiting a ceiling effect in some during the last third of the task (phase 3). This observation led us to arbitrarily distinguish three phases of the same duration and focus on phase 3, where marked differences in score-dyn emerged. Hierarchical clustering of principal components, starting with principal component analysis, identified three clusters among the participants: cluster 1 was defined by an underrepresentation of score-dyn, MF, and t**_MF_**; cluster 2 was defined by an overrepresentation of MF; and, as a critical outcome, cluster 3 was defined by an overrepresentation of score-dyn and t**_MF_**. Accordingly, participants belonging to cluster 3 had the highest score-dyn and t**_MF_**. Our interpretative hypothesis is that internal interactions that adequately perform the task are reflected in a high degree of multifractal nonlinearity. These findings extend the notion that multifractal nonlinearity is a useful conceptual framework for shedding light on adaptive behavior during complex tasks.

## 1. Introduction

Computational methods derived from the fields of nonlinear dynamics and statistical physics have increasingly contributed to human biology, paving the way for a complex systems approach to physiology [1] and cognition [2,3]. These methods can quantify correlation properties, moment-to-moment features, and scaling organization in fluctuation time series. The most commonly derived statistical indicators are rooted in the concepts of fractal analysis and entropy. Researchers have adopted a convergent point of view regarding dynamical organization in physiological systems, emerging from intricate interactions with a tendency toward nonlinearity [4,5]. These systems can function and respond across a wide range of time scales. They exhibit irregular, intermittent, and context-sensitive behavior, inevitably reflected in certain degrees of irregularity in output signals [6,7]. Irregular fluctuations in movement time series are clearly observable during task-directed behavior, and the idea that the temporal structure of these fluctuations carries critical information about underlying processing mechanisms has been developed. Adequate behavior during a task relies on the adaptive capacity of a large movement system that coordinates perception, cognition, and action. This coordination relies on a myriad of internal interactions that are not directly observable. Here we adopt the conceptual point of view that interactivity begets adaptive capacity and that interactivity is reflected in the temporal structure of movement fluctuations. The direct implication of conceiving such an interaction-dominant, complex, and nonlinear function is that one’s adaptive capacity during a task is great when multiscale interactions are robust and diverse. This could be objectively assessed through the lens of the (degree of) multifractality exhibited by the output (behavior) of complex systems. To provide researchers with an appropriate metric for adaptive capacity during task performance, theoretical and experimental efforts, including multifractal analyses of synthetic or behavioral time series, have been made to show the pertinence of compressing information into a single statistical parameter that is the width of the multifractal spectrum [8].

The multifractal framework deals with a spectrum, f(α), of singularity strengths, α, where α accounts for the power-law growth (scaling) of a proportional number of bin samples as a function of bin length, P(L)∝L^α^, and f(α) accounts for Shannon entropy, (L)∝L^α^ [9]. The multifractal spectrum width is determined by the difference (α max–α min). To determine the spectrum, a set of q-moments is applied to P and Shannon entropy to accentuate heterogeneity by reshaping the sequence of bin proportions. Each q provides a different P(L)∝L^α^ relationship. The α and f(α) for a given q are considered part of the multifractal spectrum under the condition that both the loglog P/L relationship and the loglog Shannon entropy/L relationship demonstrate stable linearity [9]. Linear stability is acknowledged using an arbitrary threshold value for the correlation coefficient, r > 0.95. Reference [9] is a must-read study for interested readers, who can also find a numerical application in [10].

A certain degree of multifractality has been recurrently observed in perceptuomotor activities [4,5,11,12,13,14,15]. However, the multifractal spectrum width accounts for both the linear and nonlinear temporal structures present in behavioral series and thereby cannot distinguish the degree of two forms of internal system interactivity: scale-dependent interactions, or alternatively across-scale interactions. Still, researchers in the literature commonly assume that nonlinearity reflects a critical property of interactivity within the system, closely linked to adaptive capacity [16]. For a deeper analysis allowing us to access the degree of nonlinearity in behavior, the multifractal (MF) width of the original series can be compared with the MF width of linearized surrogates of the same series [7,9]. This comparison provides a t-statistic (t**_MF_**) representing a continuous descriptor of the degree of nonlinearity in the measured time series.

Here, we adopt the conceptual framework that the adaptive capacity during a complex task execution relies on a certain degree of nonlinear, across-scale interactions in the movement system, enabling the flexible coordination of perception, cognition, and action. The main new hypothesis is that multifractal nonlinearity during a demanding task is preserved as long as the task is successfully performed. As an experiment, we implemented a herding task on a computer: the participant had to keep three moving sheep trying to escape a circular pen as close as possible to the center of the screen by manipulating the mouse pointer as a repelling sheepdog. The task is pleasant enough, with a scoring incentive, to promote engagement for several minutes [11,17,18]. Random sheep displacements are guided by the amplitude of a random Perlin noise. This amplitude was defined proportionally to the progression of the participant’s score to generate an individual rate of increase in task difficulty. We expected data-driven difficulty with a possible score ceiling when one’s adaptive capacity reaches a plateau as an opportunity to link the concomitant nonlinear multifractal structure of behavior.

## 2. Materials and Methods

### 2.1. Participants

Twenty-one healthy young athletes (aged 18–30, 7 women, 11.85 ± 5.71 h of sports per week), all members of the faculty of sports sciences, gave their written consent to take part in this study, which was approved by the Institutional Review Board of the “Faculté des STAPS”. The procedures followed the ethical recommendations and the rules of the Declaration of Helsinki. Ineligibility criteria included uncorrected visual impairment. Participants were instructed to avoid alcohol and caffeinated beverages for 12 h prior to the session and to refrain from strenuous physical activity the day before.

### 2.2. Procedure

Upon arrival at the laboratory, participants sat in front of a 31.5-inch (80 cm) 1920 × 1080-pixel screen positioned 70 cm away (ProLite TF3222MC, iiyama, Hoofddorp, the Netherlands) and used a computer mouse to complete a series of computerized questionnaires, taking about 8 min. The questionnaire provided a period that enabled them to settle in, situate themselves, and orient to the experimenters and the experimental environment, allowing all participants to be in a similar state before the start of the test. Afterward, they completed a familiarization phase with the herding task and the entire experimental setup (4 min). Then, they performed the herding task for 7 min in a quiet environment without any other interactions.

### 2.3. Experimental Setup

**Figure 1:**
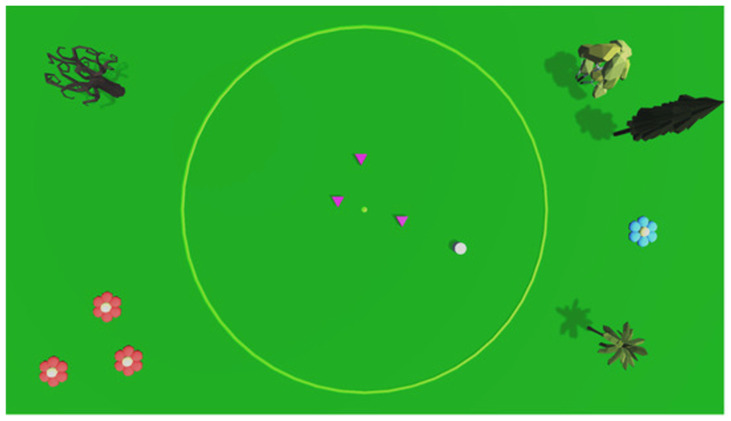
Screenshot of the herding task, inspired by Bennett et al. [11]. The pen is represented by a circle (diameter, 17 Unity units, which means 0.35 m under our conditions). The sheep are the magenta triangles, and the dog (cursor position) is the circle. The aim is to gather the sheep at the center of the pen and not to let them out during the 7 min task (see details in Section 2.3.1). The additional elements (flowers, trees) are only of aesthetic interest here.

We used a custom version of a herding task (Figure 1) developed within the Unity4 framework (version 2021.3.16f1, Unity Technologies, San Francisco, CA, USA), inspired by Bennett et al. [2]. The task required participants to prevent three “sheep” from leaving a circular pen by controlling the mouse pointer, the cursor, as a “dog”. Each sheep exhibited continuous planar displacements driven by a combination of three forces: (1) Cohesion force: This force attracted each sheep toward the barycenter of the flock (i.e., the average position of all sheep). The magnitude of this attractive force was fixed at 3 units. (2) Repulsion force: This force pushed each sheep away from the dog. Its magnitude depended on the distance between the sheep and the dog: it was null when the distance exceeded 10 distance units and increased progressively as the dog–sheep distance decreased, reaching up to 32 units if the distance reached zero. Hence, this repulsive force can reach a 10-fold higher value than the Cohesion force. (3) Random force (Perlin noise): To simulate natural, smooth, and continuous trajectories, a two-dimensional Perlin noise vector was permanently applied to each sheep. The choice of Perlin noise, inherently characterized by smooth changes, was dictated by user experience; it enabled structured motor planning and continuous adjustments by supporting perceptual-motor coordination. At each time step in sheep displacement, the Perlin noise generated a two-dimensional (planar) force vector, with each component (on the X- and Y-axes) positive or negative and ranging from 0 to 16.5 units at task initialization. For every 1000 points earned, the amplitude of this interval increased by 1.5 units on each axis, which was the main means by which the difficulty increased (as a function of the score).

#### 2.3.1. Scoring System and Task Difficulty

The score increased when the sheep remained within the pen, proportional to the distance from the center. The overall score is the sum of each sheep score.

The formula to calculate the score for one sheep was

(1) score_sheep_ = i_distance_·(level + 1)/3

where i_distance_ is the distance index of the sheep from the center of the circle, varying from 10 (sheep at the center) to 0 (sheep on the pen), and level represents the level reached by the participant: 1 level = 1000 points. The score earned each second is the sum of the three individual score_sheep_ values_._

If a sheep exited the pen, the score decreased (five points lost every second per sheep outside of the pen). If a sheep moved too far from the pen, it was reset to the center, and the score dropped to the nearest thousand. To dynamically adjust the task difficulty, the Perlin noise intensity increased for every thousand points earned, making the task progressively more challenging. This adaptive scoring system ensured that difficulty increased until some (but not all) participants reached their performance limit, at which point, the score is expected to plateau (the difficulty level cannot decrease). We chose a task-driven design in which the difficulty adapted to each participant’s performance level to maintain engagement and prevent frustration.

#### 2.3.2. Cursor Displacements

We obtained the time series from cursor displacements on the screen during the 7 min task, with a 50 Hz sampling rate triggered by the Unity System. This resulted in each series comprising 21,000 samples. The first 90 samples were discarded because initial cursor displacements were not pertinent when the participant just clicked to start the task. The cursor’s time series were obtained by calculating the Euclidean distance between two consecutive points in the Cartesian plane (x and y):

(2) $$\sqrt{{\left(x_{i+1}-x_{i}\right)}^{2}{+\left(y_{i+1}-y_{i}\right)}^{2}}$$

where *i* indicates the sample range in the series, and x and y are the horizontal and vertical coordinates of the cursor on the screen, respectively. We arbitrarily divided the cursor’s time series into three phases with an equal duration (6970 samples). Null values were discarded, as they indicate a brief absence of movement, representing an average of 6.5% of the samples in each time series.

We also calculated the average cursor speed and an entropy marker of displacement series using the Refined Composite Multiscale Entropy (RCMSE) method [19]. With RCMSE, sample entropy (SaEn) was obtained for 30 time scales after coarse-graining. The overall entropy information was compressed into a single index by calculating the trapezoidal numerical integration of the SaEn values over the 30 scales.

### 2.4. The Multifractal Spectrum Width

**Figure 2:**
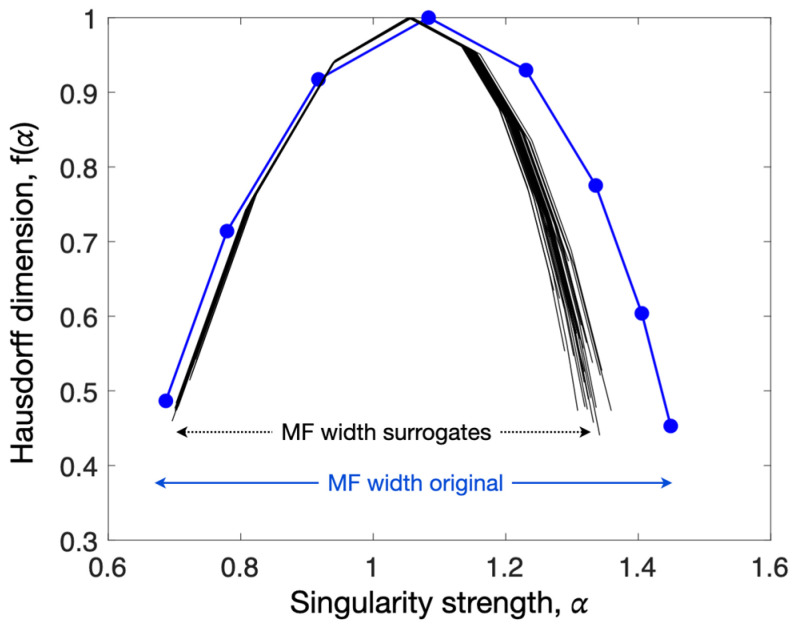
The multifractal spectrum of a typical time series (blue line with dots) and the multifractal spectra obtained by the 40 linearized surrogates of the same series (black lines). The high MF width of the original series compared with the MF widths of the IAAFT surrogates (n = 40) suggests multifractal nonlinearity, quantified by a t-statistic value (t**_MF_**).

We directly estimated multifractal spectra that characterized the cursor time series in each phase using Chhabra and Jensen’s analytical method [8]. The multifractal spectrum represents the relationship between two fractal dimensions called α and f(α) (see Figure 2). The method is based on Shannon entropy, which is well suited for characterizing multiplicative cascades. A comprehensive analysis is available elsewhere [9]. In short, the analyzed series is iteratively sequenced in non-overlapping bins, where the number of samples in each bin (or bin length, L) also represents the observational time scale. Then, bin proportion, P_i_(L), is calculated by dividing the sum of bin samples by the sum of the samples of the whole series. After that, the measured series is deformed using a set of q exponents that amplify either large-sized fluctuations (when positive q values are used) or small-sized fluctuations (negative q values). A simple numerical example is provided in Appendix A of [10].

The multifractal spectrum is defined by pairs of f(*q*,*L*) and α(*q,L*), obtained after a mass coefficient (μ_i_) is calculated for each observational scale and q moment:

(3) $$\mu_{i}\left(q,L\right)=\;\frac{{\left[P_{i}(L)\right]}^{q}}{\sum_{i}{\left[P_{i}(L)\right]}^{q}}$$

The singularity strength, α(*q*), is the singularity for the μ(*q*)-weighted *P(L)*, estimated by

(4) $$\alpha (q)=\underset{L\to 0}{\lim}\frac{\sum_{i}\mu_{i}(q,L){logP}_{i}(L)}{logL}$$

The estimates, α(*q*), belong to the multifractal spectrum if the Shannon entropy of μ(*q,L*) evolves with L along a dimension, f(*q*), calculated as

(5) $$f(q)=\underset{L\to 0}{\lim}\frac{\sum_{i}\mu_{i}(q,L){log\mu }_{i}(q,L)}{logL}$$

The q-order generalization of the singularity strength, α, is the slope of the μ-weighted P against L on a loglog scale. The q-order generalization of the Hausdorff dimension, f, is the slope of the Shannon entropy (the negative Shannon entropy) against log L. By considering the paired value of α and f at the given q value, the multifractal spectrum is obtained by plotting f(*q*)~α(*q*). Here, we used a range of q values [−10:10]. To establish the final multifractal spectrum, it is advisable to hold only q values that achieve relative stability in the μ(*q*)log P~log P and μ(*q*)logμ(*q*)~log P linear relationships. This is achieved by considering a benchmark as a threshold—an r^2^ > 0.9 in linear fits here—below which, linearity is not an acceptable model for quantifying α and f.

The main variable retained in the present analysis is the multifractal spectrum width, MF. MF was obtained by calculating the difference between the higher and lower values of the singularity strength, α (Figure 2).

#### 2.4.1. Linearized Surrogates Obtained with IAAFT

To address the question of nonlinearity that is central to our approach, it is advisable to build series that mimic the best-fitting linear model of the original series. For that, a procedure was developed by Schreiber and Schmitz [20], and recommended by Ihlen and Vereijken (2010), to test whether multifractality in the original series reflects nonlinear interactions across time scales. The iterative amplitude-adjusted Fourier transform (IAAFT) procedure is used to generate a series of the same measured samples as in the original series, put in a different order. The procedure is detailed in [9]. In brief, the list of amplitudes identified in the Fourier Transform spectrum of the original series is preserved, but the correspondence with the list of available frequencies is randomized. Given that a linear model will hold irrespective of the ordering, nonlinearity is revealed by a difference in the multifractal spectrum width of the original series and the multifractal spectrum width and its surrogates. Therefore, to characterize the nonlinearity of our signals, we applied the analytical method of Chhabra and Jensen (described in 2.4) to our surrogates’ series.

#### 2.4.2. Nonlinear Multifractality: Surrogate Data Testing

Linearization in surrogates eliminates nonlinear features initially present in the measured series. Thus, the MF spectrum of the original series must be larger than the MF spectra of the surrogate series (Figure 2). Kelty-Stephen et al. [9] suggest a one-sample *t*-test to compare the original series’ multifractal spectrum width to the width of a finite set of linearized surrogates with a matching linear structure. This way, the t-statistic value (t**_MF_**) provides a continuous marker of the difference from the surrogates that quantifies how much the original series’ multifractality departs from the multifractality attributable to linear structures. Here, we tested if t**_MF_** in this comparison amounted to a low or high value for each behavior. For that, we subtracted the MF width in every 40 surrogates from the MF width in the original series, using a one-sided *t*-test to test the null hypothesis that the data comes from a population with a mean equal to zero or the alternative hypothesis that the data comes from a population with a mean greater than zero. t**_MF_** served as an estimate of the degree of multifractal nonlinearity in behavior in subsequent analyses.

### 2.5. Statistics

#### 2.5.1. Between-Phase Comparisons

Normality was tested for each analyzed variable using the Shapiro–Wilk test. As the task was divided into three phases, comparisons between phases were performed with the Friedman test. For post hoc tests, we applied the Conover correction for multiple comparisons to control for the potential increase in Type I errors.

#### 2.5.2. Factor Analysis of Data

We performed a multivariate analysis by first performing a principal component analysis (PCA), followed by hierarchical clustering of principal components (HCPC) to identify clusters based on PCA-derived dimensions. PCA characterized the association between the main variables obtained in phase 3 of the task: score-dyn, MF, and t**_MF_**. We introduced additional variables to the PCA map, assessing the weekly physical training load of the participants and other characteristics of the cursor’s dynamics (speed, idle time, and RCMSE) to observe possible covariations. The hierarchical tree considered used Ward’s criterion, which consists of a series of agglomerative steps. In brief, participants were successively combined into clusters, which maximized both internal homogeneity, i.e., within-cluster variation, and external heterogeneity, i.e., between-cluster variation. According to Ward’s HCPC method, each emerging cluster is determined by the least increase in the sum of the squared Euclidean distances, which reduces the inevitable loss of information associated with cluster fusion. Simply put, the main information returned by HCPC lies in both the position of a participant or cluster of participants with respect to the *x*- and *y*-axes—which represent specific dimensions of the PCA—and the v.test parameter of a cluster, which indicates the variable with the most important weight in characterizing that cluster.

The data were analyzed in JASP (version 0.17.1.0, https://jasp-stats.org, accessed on 12 April 2023) and R (R Core Team (2020); R: A language and environment for statistical computing; R Foundation for Statistical Computing, Vienna, Austria).

## 3. Results

### 3.1. Score Dynamics During Each Phase

**Figure 3:**
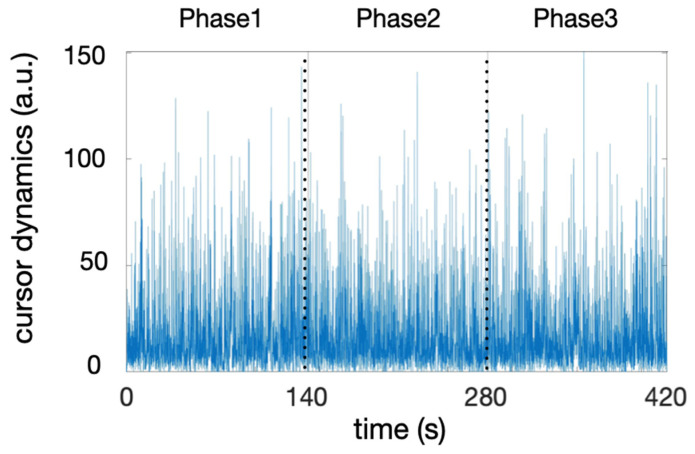
A typical time series of cursor dynamics during the 7 min herding task for one participant. Three phases of equal duration are delimited by dotted vertical lines.

**Figure 4:**
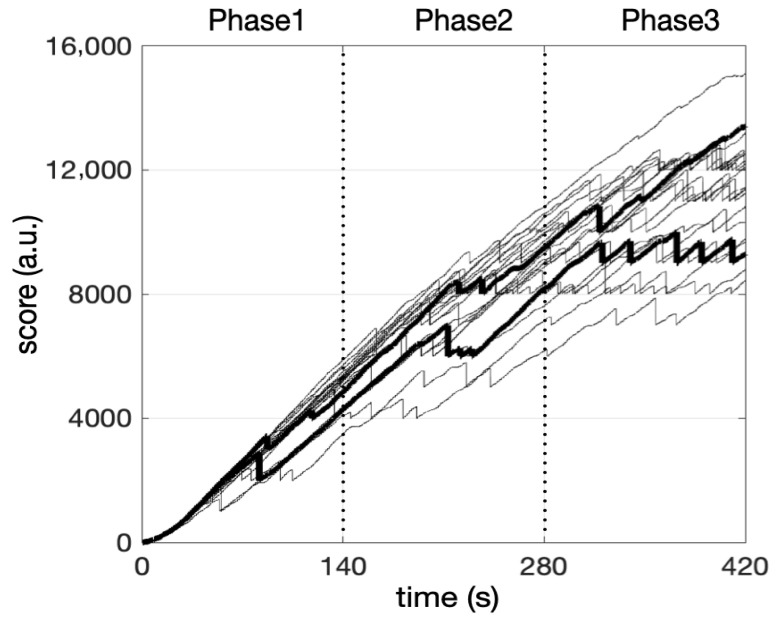
Time course of the score for all participants throughout the task. Three phases of equal duration are indicated by vertical dotted lines. The score increased depending on the participant’s ability to keep the sheep inside the pen. Difficulty increased every 1000 points, increasing Perlin noise amplitude and, thus, difficulty. For example, reaching 8000 points meant that the participant had crossed eight steps in the Perlin noise scale. The sharp drop (drop to the nearest 1000) in scores illustrates a penalty due to the sheep escaping the pen. Bold lines show two typical participants exhibiting either a preserved score increase or a ceiling effect in phase 3.

Figure 3 illustrates the cursor time series of one participant as an example and shows the arbitrary three phases of the task. We collected the maximum score (scoreMax) achieved in each phase. Phase-specific score dynamics were obtained from linear fit slopes of score vs. time. The data for the score dynamics and the maximum score do not follow a normal distribution. A Friedman test on the scoreMax revealed a significant effect across phases (χ^2^(2) = 42.00, *p* < 0.001). This means that all subjects continued to progress across the three phases. This is supported by the Conover post hoc test, which reveals a significant difference between each phase (*p* < 0.001 for all pairwise comparisons). As the time spent on the task increases, the standard deviation of the maximum scores achieved during each phase also increases. This indicates growing variability in individual performance, suggesting that personal strategies become more unevenly effective as task difficulty rises. The Friedman test showed different score dynamics values across phases: χ^2^(2) = 42.00, *p* < 0.001. Post hoc analyses using the Conover test showed significant differences in score-dyn between phases 1 and 2 (t = 3.24, *p* = 0.002), between phases 2 and 3 (t = 3.24, *p* = 0.002), and between phases 1 and 3 (t = 6.48, *p* < 0.001). The rate of score increases decreased throughout the task, confirming the progressive task complexity. This decline was particularly pronounced between phases 2 and 3, where the average slope resulted from a plateau in some participants and a further improvement in performance in others, as illustrated in Figure 4. This heterogeneity is reflected in coefficients of variation that were 2-to-3-fold higher in phase 3 (see Table 1).

### 3.2. Multifractal Nonlinear Metrics of Behavior: MF and t**_MF_**

The participant’s behavior during the task was represented by the dynamics of the cursor displacements, as calculated with Equation (1) and illustrated in Figure 3. Behavioral complexity (irregularity) was quantified by two variables, the multifractal spectrum width (MF) and the t-statistic (t**_MF_**), which were obtained by comparing the MF of the original series and the MF of 40 linearized surrogates obtained using IAAF (Figure 2). The phase-specific values of MF and t**_MF_** are reported in Table 1.

As the t**_MF_** distribution did not satisfy the assumption of normality, a non-parametric Friedman test was performed. No significant difference between phases was observed (χ^2^(2) = 1.81, *p* = 0.405). For consistency in the statistical analyses, a Friedman test was also performed for the MF data, which indicated no significant effect for time (χ^2^(2) = 4.67, *p* = 0.097).

### 3.3. Hierarchical Clustering on Principal Component

**Figure 5:**
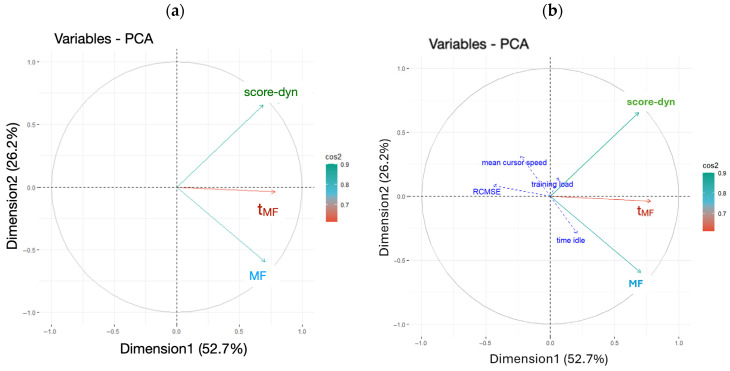
Principal component analysis with the two main dimensions projected on orthogonal axes. PCA is illustrated in (**a**) (the main factors) and (**b**) with additional variables.

**Figure 6:**
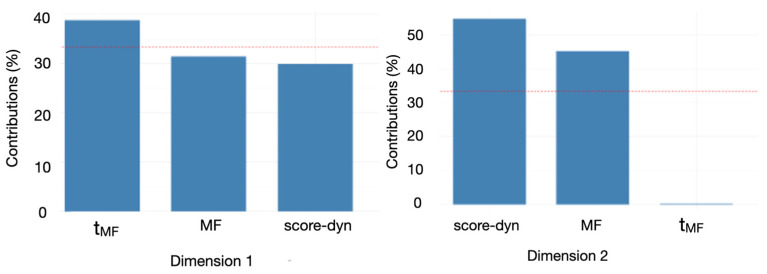
Contribution of each variable to the first two dimensions of the PCA, illustrated by orthogonal axes. The dotted line indicates the average expected contribution (100/number of variables). Variables above this threshold contribute more than average to the component. Thus, in the PCA in Figure 5, Dimension 2 is mainly described by both score-dyn (upper range) and MF (lower range); Dimension 1 is mainly described by t**_MF_**.

**Figure 7:**
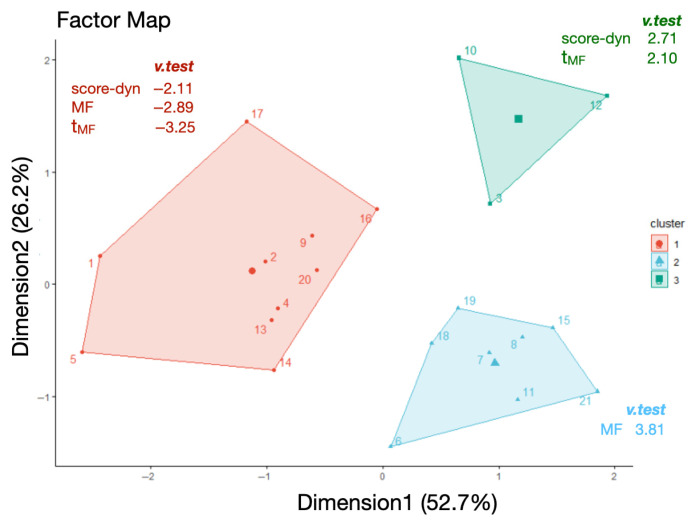
Mapping of the clusters as a function of PCA axes seen in Figure 5. Participants are numbered and colored according to their cluster. The barycenter of each cluster is represented by a larger symbol. v.test represents the statistical value used to determine the significance of the variables describing the group (a positive value indicates an overrepresentation of the modality under consideration; a negative value represents an underrepresentation).

It is obvious that during phase 3, the score of some participants is close to stagnant, as quantified by low score-dyn values, while others preserved a significant increase (high score-dyn value). Thus, phase 3 served as a basis to establish a potential link between the multifractal markers of adaptive behavior and the ability to overcome task difficulty. Thus, score-dyn, MF, and t**_MF_** were subjected to a Hierarchical Clustering on Principal Component (HCPC) analysis comprising two steps. First, a principal component analysis (PCA) was computed to select the two main PCA dimensions and to determine the main contributors for each dimension (Figure 5). From the PCA, we retained two main factors, Dimension 1 and Dimension 2, which explained, respectively, 53% and 26% of the total variance (Figure 6). The most weighted contribution to Dimension 1 was multifractal nonlinearity, t**_MF_** (Figure 6). The most weighted contributions to Dimension 2 were score-dyn and MF. Then, the HCPC was performed to delineate clusters of participants with similar characteristics (Figure 7).

The HCPC returned a set of three clusters (Figure 7). Each participant was positioned on the factor map (PCA) within the cluster they belonged to. Thus, individual positions in relation to Dimensions 1 and 2 can be visualized. The individual position of each participant in relation to the orthogonal axes indicates the respective contributions of t**_MF_**, the main contributor to Dimension 1, as well as score-dyn (up) and MF (down), the main contributors to Dimension 2. For further analysis, the most weighted variables in each cluster are identified by the v.test value (Figure 7).

In cluster 1, the three variables score-dyn, MF, and t**_MF_** are underrepresented, as indicated by the negative v.test values for each variable (Figure 7, red cluster). Thus, participants in cluster 1 have rather poor score dynamics during phase 3 and exhibit rather low MF and t**_MF_** values, especially in the v.test value (−3.25) and the position of most participants along Dimension 1 with the main contributor, t**_MF_**. This indicates that t**_MF_** is a weak point in these participants. In cluster 2 (in blue), the most weighted variable was the width of the multifractal spectrum of behavior, MF. Although most of the participants in cluster 2 are positioned on the right of Dimension 1 with the main contributor, t**_MF_**, t**_MF_** was not specially weighted in this cluster. Interestingly, the best performers during phase 3 pertain to cluster 3 (in green). The two most weighted variables in cluster 3 were score-dyn and t**_MF_**, meaning that cluster 3 has grouped three participants who preserved their performance (score-dyn) during phase 3 of the task, exhibiting high multifractal nonlinearity in their behavior over the same period. Additional variables in the PCA (Figure 5b) provided no additional information.

The cluster-averaged values of each variable are indicated in Table 2.

As t**_MF_** comes out as a central indicator of behavioral coordination, we explored the relationship between t**_MF_** and motor variability, degree of irregularity (RCMSE), and final performance (maximum score achieved). Spearman’s rank correlation showed no correlation between t**_MF_** and these indicators. This suggests that t**_MF_** captures specific behavioral aspects that are not reflected in other metrics.

## 4. Discussion

The main finding of the present study is the possible link between an individual’s adaptive capacity to overcome the difficulty of a complex computer task and the degree of nonlinear multifractal structure in behavior. Exploiting the movement system as a complex dynamical system, we used the width of the multifractal spectrum of behavior and, more specifically, the degree of nonlinearity to account for the adaptive capacity to overcome progressive difficulty during a computer task that involves controlling the mouse pointer.

Under our conditions, all participants exhibited multifractal behavior reflected in the cursor’s displacements during phases 1, 2, and 3 of the herding task (Table 1), wherein difficulty increased progressively at an individual task-driven rate. This demonstrates that the mathematical language that considers bin proportion, P, and the Shannon entropy of the bin proportion in relation to the bin size, L, provides a consistent view of task-directed behavior; in a single statistical estimate, it can compress the multifractal spectrum width, MF. However, the most striking observation is that despite progressive difficulty as the task progressed, MF width did not vary from the earliest (phase 1) to later stages (phase 3), despite ceiling effects observed in phase 3. Clearly, the absence of change in MF throughout the task contrasts with more variable estimates of the overall ability of the participants to succeed in sheepherding, reflected in the time course of the score, score-dyn (Table 1). Indeed, score-dyn decreased throughout the task as a result of a rise in difficulty and, more importantly, varied considerably across participants in phase 3, where ceiling effects in some participants contrasted with preserved increases in score in others. Since MF and score-dyn did not covary, MF per se could be blind to task difficulty and the necessary adaptations under our conditions. Comparatively, the analysis of multifractal nonlinearity, t**_MF_**, provided better matching with the way score increases are preserved or not for each participant. At the group level, both t**_MF_** and score-dyn reached their lowest values in phase 3, and more importantly, both t**_MF_** and score-dyn exhibited their highest interindividual variability (Table 1). This might underline a distinctive adaptive capacity among the participants at this particular stage. The multivariate analysis made a significant contribution to understanding the possible links between multifractal nonlinearity reflected in t**_MF_** and adaptive capacity reflected in score-dyn, as well as the superior reliability of t**_MF_**. First, the cluster analysis applied to phase 3 showed that most participants with low MF also had a low score-dyn (cluster 1). This might provide a first-level indication that a low degree of multifractality in behavior does not represent an adequate internal interactivity to overcome task difficulty. However, the opposite is not strictly true, as participants grouped in cluster 2 exhibited high MF but a weak capacity to preserve score increases (score-dyn) during phase 3. Thus, the present multivariate analysis might indicate that MF, per se, is useful for excluding individuals with poor adaptive capacity but is not sensitive enough to finely distinguish a superior adaptive capacity to overcome task difficulty. More interestingly, and as a primary finding, t**_MF_** seemed to be much more sensitive to the successful execution of the task, as cluster 3 grouped participants who exhibited a strong covariation between t**_MF_** and the preserved capacity to increase the score (Figure 6). This result aligns with the notion that critical processing for adaptive perceptual–motor coordination emerges from nonlinear interactions in the perceptual–motor system.

Although a certain perspective on cognition posits that individual system components operate independently within a causal chain—such that adaptive behavior can be attributed to a specific element of this chain—this view remains limited. Here, we support the emerging idea that multifractal nonlinearity, as assessed, for instance, by t**_MF_**, reflects a critical internal organization of the system that underlies its adaptive capacity.

Aligning with the present experiment, the complex system, fractal-based approach of adaptive behavior, or altered behavior, has brought about significant advances in the last twenty years. The monofractal approach, dedicated to continuous rhythmic behavior, has highlighted the presence of 1/f noise when external constraints do not dominate the internal constraints of mental running systems during cognitive task performance [2]. Fractal dynamics also dominates in the absence of pathology during task-directed movements of the upper [21] or lower body [22]. Sensory input deprivation is also reflected in the fractal behavior of the system [23], as well as the difficulty of a task [24]. Multifractal analyses are dedicated to intermittent behavior, generally exhibiting not a singularity but a superimposition of fractal behavior, which is illustrated in the present study by the range of singularity strengths (Figure 2). Multifractal evidence of complex processes within the perceptual–motor system, resembling multiplicative cascades [25], has consistently informed adaptive behavior. This reveals the importance of across-scale interactivity in upright postural control; before the introduction of multifractal nonlinearity, this was mainly considered a scale-dependent control, where insular, short-time feedback events and top-down, long-time pro-active actions co-exist but operate in distinctive, scale-independent ways [13,26,27,28]. Multifractality has also shed new light on cognition, where different types of interactivity support executive functions [4,5,15]. Assessing the degree of multifractality and nonlinear multifractality also provides a deeper insight into perceptual–motor responses [14,29,30,31,32,33,34], with clear implications for understanding deficiencies [35,36,37] and the development of rehabilitation programs [38]. Previous uses of multifractality in mouse control during herding tasks have also grounded the approach in the human–computer interface (HCI) field. This allows cognition to track tool readiness-to-hand [11,18] and a participant’s engagement in computer tasks. By introducing t**_MF_** as an index that covaries with the ability to perform a task requiring the flexible use of visual search, planning, and attention, we aimed to provide an additional tool for a conceptual approach that assumes critical roots in cognition and behavior grounded in multifractal nonlinearity.

The present study has some limitations and interesting perspectives. Although the multifractal spectrum is sometimes asymmetric (Figure 2), here, we paid no attention to asymmetry in the MF spectra of the empirical series. Asymmetry has possible origins in subtle coordination across cascading processes [25], which could help decipher the links between cognition and other important functions [10]. The use of HCPC was motivated by profiling individual behaviors. For an exhaustive exploration, a larger sample of participants may be more appropriate. We limited the number of variables introduced in the PCA and HCPC to achieve sufficient statistical power, but including more participants could help decipher the potential role played by other factors. In the same vein, the participants were all young and healthy adults, which prevents generalization to other populations.

## Figures and Tables

**Table 1:** Mean values ± standard deviations and ranges (in brackets) of main variables in each phase of the task. Although score-dyn and t**_MF_** showed non-normal distribution, mean and sd are presented for simplicity.

Variable	Phase 1	Phase 2	Phase 3
score-dyn	38.1 ± 5.6 * [22.3:45.2]	27.3 ± 6.2 * [14.8:37.8]	16.0 ± 7.2 * [2.7:31.8]
MF	0.58 ± 0.07 [0.41:0.74]	0.60 ± 0.08 [0.45:0.77]	0.60 ± 0.06 [0.51:0.70]
t**_MF_**	8.8 ± 10.8 [−8.0:31.0]	10.8 ± 11.3 [−8.1:36.3]	6.5 ± 7. 6 [−5.0:16.6]
scoreMax	5082 ± 609 * [3542:5922]	9146 ± 1214 * [6184:10,837]	11,451 ± 1650 * [8427:15,108]

* indicates differences from each of the other phases.

**Table 2:** Mean values and standard deviations of main variables in each independent cluster.

Cluster Id.	Score-dyn	MF	t_MF_
Cluster 1	12.5 ± 5.6	0.56 ± 0.04	0.88 ± 5.10
Cluster 2	16.4 ± 5.4	0.67 ± 0.03	10.34 ± 6.00
Cluster 3	26.4 ± 5.8	0.57 ± 0.04	14.97 ± 1.90

## Data Availability

The datasets are available from the corresponding author upon reasonable request.

## Abbreviations

The following abbreviations are used in this manuscript:

MF	Multifractality; MF designates the width of the multifractal spectrum
tMF	A t-statistic value that indicates the degree of multifractal nonlinearity
score-dyn	This indicates the score dynamics, the rate of change in the score (evolution over time of the number of points gained and lost by the subject during the task) as a function of time
